# Impact of dynamic beam delivery of intensity-modulated radiation therapy and volumetric modulated arc therapy on circulating immune cell exposure in glioma

**DOI:** 10.1016/j.phro.2026.101070

**Published:** 2026-08-25

**Authors:** Abdelkhalek Hammi, Nadya Shusharina, Eleni Gkika, Youness Nour

**Affiliations:** a Physics Department, TU Dortmund University, Dortmund, Germany; b Division of Radiation Biophysics, Department of Radiation Oncology, Massachusetts General Hospital, Boston, Massachusetts, USA; c Harvard Medical School, Boston, Massachusetts, USA; d Department of Radiation Oncology, University Hospital Bonn, University of Bonn, Bonn, Germany

**Keywords:** Circulating blood cells exposure, Radiation-induced lymphopenia, Dynamic beam delivery, IMRT & VMAT, Glioblastoma

## Abstract

**Background and purpose:**

To compare how intensity-modulated radiation therapy (IMRT) and volumetric modulated arc therapy (VMAT) affect circulating blood cells (CBC) depletion in glioma patients.

**Materials and methods:**

Twenty-eight glioma patients were retrospectively analyzed. Mean clinical target volume (CTV) was 293.8 ± 152.4 cm^3^ (μ±σ). For each clinical IMRT or VMAT plan, a corresponding plan using the alternative technique was generated with identical objectives and constraints. A 4D dynamic blood-flow model simulated cerebral blood flow and time-resolved dose delivery to estimate accumulated dose and CBC depletion. Endpoints included irradiated blood volume (V_D>0_) and CBC depletion relative to baseline. Linear mixed-effects modeling assessed technique, CTV volume, and their interaction.

**Results:**

After one fraction, mean V_D>0_ was higher with IMRT than VMAT (49.9% ± 7.2% vs 38.6% ± 6.3%; *p* < 0.00001). IMRT exposed more CBC volume in 24 of 28 patients; the four exceptions had the smallest CTVs. After three fractions, approximately 90% of CBCs were exposed with IMRT versus 77% with VMAT. Estimated CBC depletion was 13.9% with IMRT and 12.8% with VMAT (mean paired difference, 1.0 ± 1.7 percentage points; 95% CI, 0.3–1.7; *p* = 0.003). Depletion increased with CTV volume for both techniques, with a technique-by-CTV interaction of −0.41 percentage points per 100 cm^3^ (*p* = 0.041).

**Conclusions:**

IMRT and VMAT produced different blood dose distributions. However, the mean depletion difference was small relative to estimated intersubject variability and does not establish a clinically meaningful advantage for either technique.

## Highlights

• On average, Intensity-modulated radiotherapy exposed 49.9% of blood in one fraction.
• Volumetric modulated arc therapy exposed 38.6% of blood in one fraction.
• Intensity-modulated radiotherapy exposed 90% of blood within three fractions.
• Mean estimated blood cells depletion differed by 1.0 percentage point.

## Graphical abstract

**Figure:**
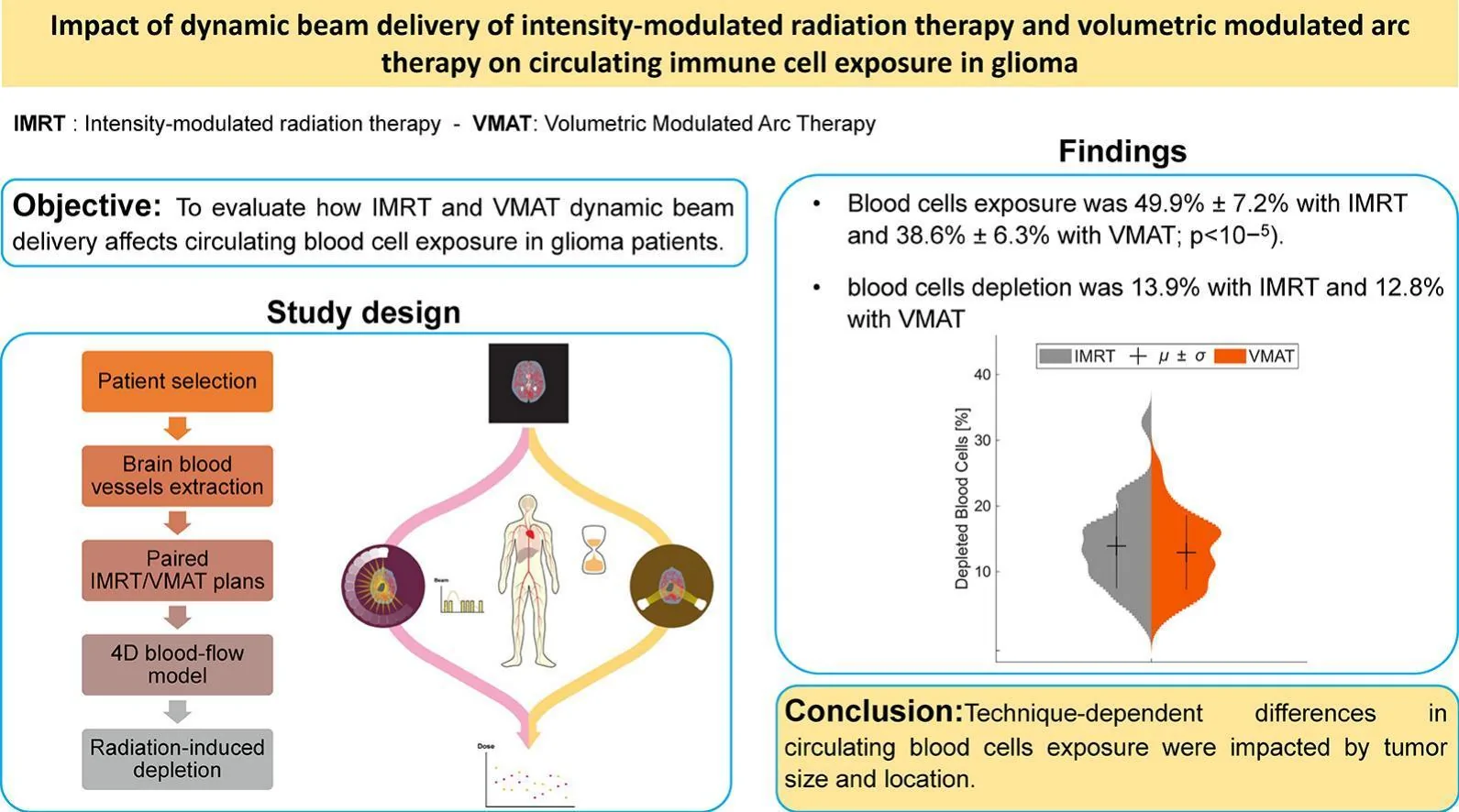

## 1 Introduction

Glioblastoma (GBM), the most common and aggressive brain tumor in adults, represents 15% of all central nervous system tumors and 50% of malignant brain tumors [1], [2]. Despite comprehensive treatments, including surgery, radiotherapy (typically 60 Gy in 30 fractions), and temozolomide, the prognosis for GBM patients remains poor, with a median survival of approximately 16 months [3], [4].

Recent clinical trials are exploring immunotherapy as an additional treatment modality for GBM [5], [6], [7], [8], [9]. However, both, radiotherapy and chemotherapy, induce lymphopenia, a lower-than-normal circulating blood cells (CBCs) level. This treatment-induced lymphopenia has been reported to correlate with poorer survival outcomes in various solid cancers, including GBM [10], [11], [12], [13], [14], [15]. Maintaining healthy CBCs level is crucial for an effective immune response against cancer. Therefore, sparing CBCs during radiotherapy may enhance the synergistic effects of immunotherapy and potentially improve outcomes for GBM patients.

Modern radiotherapy techniques, such as volumetric modulated arc therapy (VMAT) and Intensity-modulated radiation therapy (IMRT), have improved dose distribution conformality in brain treatments and reduced exposure to surrounding organs at risk, such as the brainstem, optic chiasm, optic nerves, hippocampi, and cochleae [16], [17], [18]. Despite these technical advancements, recent studies have shown that even partial brain radiotherapy can lead to radiation-induced lymphopenia (RiL) in GBM patients [19], [20]. This RiL occurred even in GBM patients treated with a reduced prescription total dose [21]. Therefore, current research focuses on further optimizing radiotherapy delivery to minimize exposure to healthy brain tissue and to develop an optimal radiotherapy that considers the radio-sensitivity of immune function [19], [20], [21].

Although radiation effects are mostly localized, inevitable exposure of surrounding lymphocyte-rich organs (e.g., skin, lymph nodes) and the out-of-field dose results in a proportion of CBCs receiving a lethal dose, particularly during fractionated treatments [22]. CBC are highly radiosensitive; even a dose of 0.3 Gy can reduce lymphocyte counts by 10% [23], a threshold that can easily be reached during fractionated radiotherapy [24]. While the immune system typically recovers a few months after treatment, 40% of GBM patients experience prolonged lymphopenia for up to a year post-treatment [25].

Currently, there is no consensus on the dose thresholds associated with RiL. The principle of lymphocyte-sparing radiotherapy has the potential to mitigate RiL [26], a notion currently under investigation in a clinical trial for non-small-cell lung cancer patients undergoing radiotherapy [27]. It is therefore crucial to understand how the dynamic beam deliveries of modern radiotherapy techniques and plan parameters (dose rates, beam directions, treatment fraction times) impact the accumulated dose to CBC and subsequent depletion, in order to optimize lymphocyte-sparing radiotherapy.

**Fig. 1:**
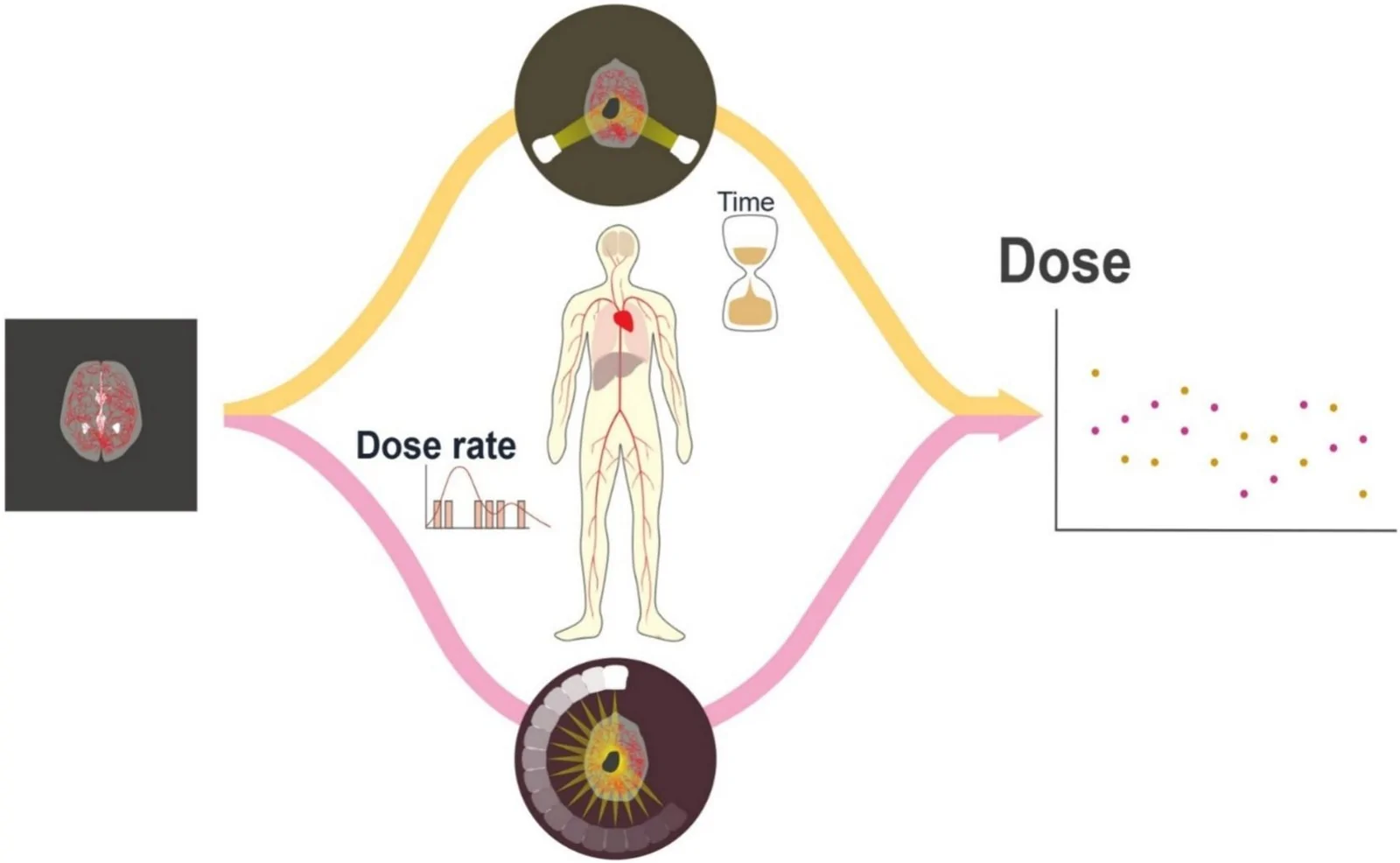
Schematic of the proposed unified 4D framework: patient-specific cerebrovascular modeling combined with dynamic beam delivery timing to simulate spatiotemporal CBC transport and compute accumulated CBC dose for IMRT (up) versus VMAT (down).

Several in silico studies have already investigated factors that affect the dose received by CBC during radiotherapy for GBM patients, including dose rate and clinical target volume (CTV) size [24], radiation type i.e. proton or photon plans) [28] and the impact of treatment time, number of segments in IMRT plans [29], and the impact of ultra-high dose rate proton radiotherapy on CBC [30]. However, comprehensive data on how IMRT and VMAT treatment plans differently impact CBC depletion in GBM are still lacking. Indeed, in IMRT, the treatment is delivered using a few radiation beams, each divided into smaller segments shaped by multileaf collimators (MLCs). The beam is turned off between segments, which makes the delivery slower and requires more monitor units (MUs) than three-dimensional conformal radiotherapy. In contrast, VMAT delivers the dose in one or more continuous arcs, using many beam angles [31], [32], [33]. This allows for faster treatment and requires fewer MUs compared to IMRT (Fig. 1). Because CBCs continuously flow through the body and the dose rate field, the longer delivery time and different numbers of MUs of IMRT- and VMAT- plans can result in different blood dose-volume histograms (b-DVHs), and thus different levels of CBC depletion [29], [30].

This retrospective study compared the effects of the distinct spatiotemporal dose-rate distributions of IMRT and VMAT plans on CBC exposure in glioma patients. A cerebral vasculature model was used to simulate time-resolved, three-dimensional (4D) blood flow, while a dynamic beam-delivery framework emulated differences in dose rate and beam-on timing between IMRT and VMAT. The effect of CTV size on the fraction of CBCs exposed during treatment and the corresponding CBC depletion relative to baseline was assessed for both modalities.

## 2 Materials and methods

### 2.1 Patient data and treatment plans

Twenty-eight patients with glioma were retrospectively analyzed using the GLIS-RT dataset [34]. The study was approved by the Massachusetts General Hospital Institutional Review Board (2023A008600). Data were transferred under a data transfer agreement.

Of these, 12 received IMRT and 16 received VMAT as their clinical treatment (Supplementary Table S1). Four patients had astrocytoma and one had oligodendroglioma. CTV volumes ranged from 57 to 725 cm^3^ with a mean of 293,8 cm^3^ ± 152,4 cm^3^ (μ±σ). Tumors were primarily unilateral, with eight cases crossing hemispheres. For systematic comparison, each patient was replanned using the alternative modality while keeping constraints and objectives identical. IMRT plans used 5–8 coplanar or noncoplanar beams with up to 25 MLC segments per beam, while VMAT plans used 1–3 arcs (see Fig. 1). All plans prescribed 60 Gy and met the prespecified target criteria of D_95%_ ≥ 57 Gy and D_10%_ ≤ 63 Gy. All plans were considered clinically acceptable by two radiation oncologists.

### 2.2 Simulation based on dynamic beam delivery

A previously validated 4D blood-flow model (BFM) was used to simulate the spatiotemporal distribution of CBC [28], [29]. This BFM contains 32 organs and is based on human hemodynamic reference data, such as organ blood volumes and flow rates, provided by the International Commission on Radiological Protection [35]. The Blood particle propagation was simulated over time using a time-discrete Markov chain approach [29], [30].

The BFM's transition matrix was optimized to reproduce a dilution curve that closely matches a published peripheral blood dilution curve measured in a healthy subject injected with Radiophosphorus P^32^-labeled blood [36]. A detailed description of the BFM and the customization of its transition matrix is provided in [28], [29].

**Fig. 2:**
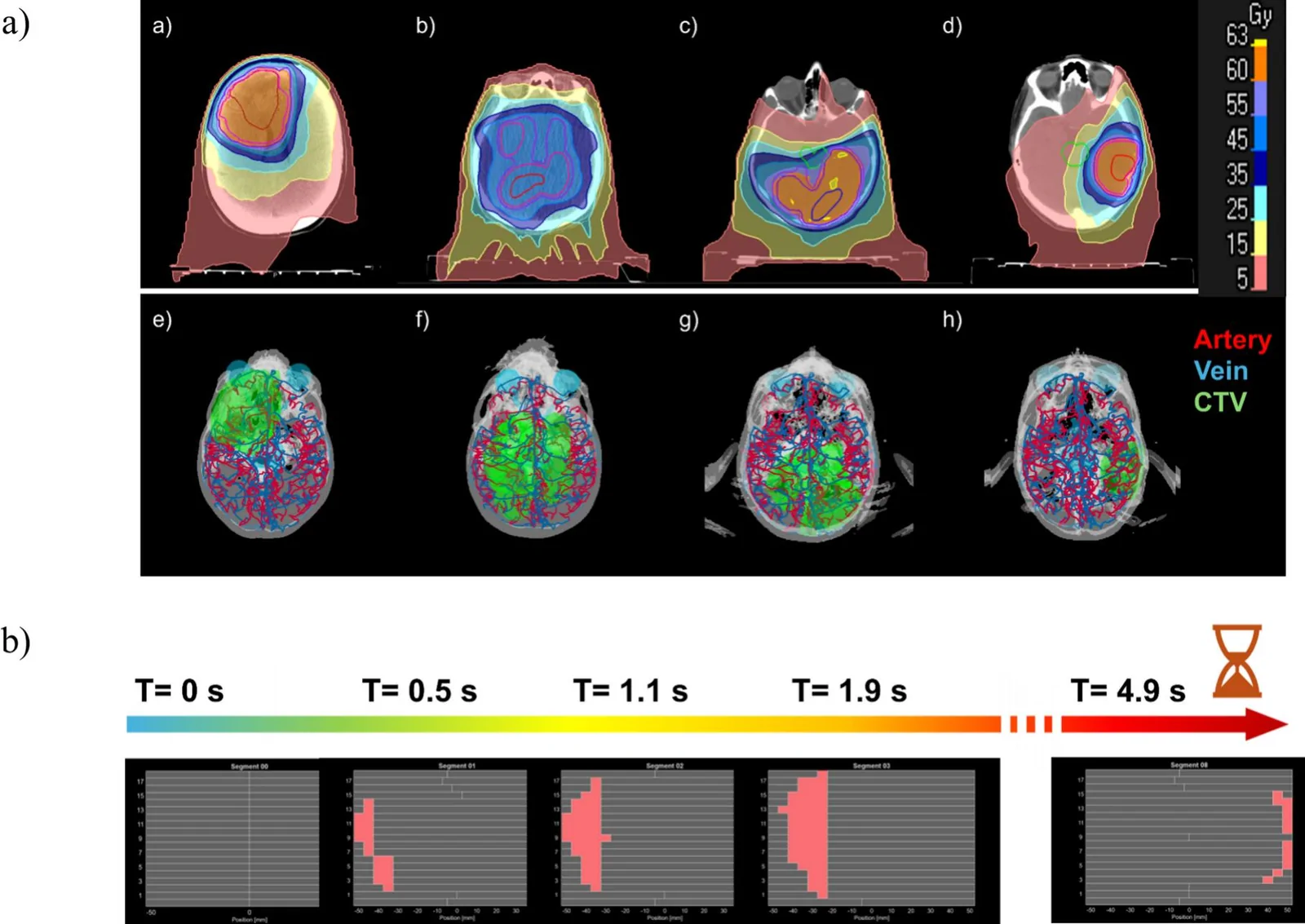
A) Upper Panel: Dose distributions for four patients (IDs: 9, 18, 23, 25), from left to right. Lower Panel: Cerebrovascular model for the same patients. Arteries are shown in red, veins in blue, and the CTV is in green. B) The time evolution of MLC openings for IMRT Field 01 (Segments 01–08) of patient 1 showing the dynamic beam shaping across segments. (For interpretation of the references to color in this figure legend, the reader is referred to the web version of this article.)

To resolve dose deposition within the brain, where the instantaneous dose rate is greater than zero ($\dot{D}\left(t\right)>0$), a hybrid cerebrovascular phantom was integrated into the model to explicitly simulate the continuous flow of blood particles through the $\dot{D}\left(t\right)$ field [30] (Fig. 2a). Major arteries and veins were segmented from 3 T magnetic resonance angiography scans and combined with fractal-based synthetic vessel trees generated using vascular territory approach [37]. The vascular structures were co-registered to each patient's planning computed tomography using 3D Slicer [38]. The simulations included an average of 20.7 × 10^6^ ± 2.1 × 10^5^ blood particles per patient, with a temporal resolution of 0.001 s.

IMRT and VMAT delivery time structures were modeled explicitly. IMRT fields followed a step-and-shoot sequence with MLC motion at 2.7 cm/s and beam-off intervals between segments (Fig. 2b). For VMAT delivery, continuous gantry rotation at up to 6°/s was assumed, with dose-rate and MLC-position modulation as a function of gantry angle. Gantry rotation, couch adjustment times, and beam-off intervals were taken into account during the blood-flow propagation to ensure synchronization between CBC trajectories and instantaneous dose rate in the patient.

All non-zero dose values in the treatment plan were included without applying an in-field mask or excluding extracranial regions. Consequently, all non-zero contributions from out-of-field scatter represented in the dose distribution were included. These comprised extracranial compartments such as the thyroid, branchial tissue, and the internal carotid arteries and jugular veins in the neck.

During radiotherapy delivery, the accumulated dose to each CBC *i* was calculated by tracking its trajectory through the spatially varying instantaneous dose-rate field, $\dot{D}\left(\vec{x},t\right)$:

(1) $$d_{i}^{F_{n}}=\sum_{j=1}^{F_{n}}\sum_{t=0}^{T_{F_{j}}}\dot{D}\left(\vec{x},t\right)*∆t$$

where $F_{n}$ is the fraction number. $T_{F_{j}}$ is the treatment time of the *j-*th fraction and ∆t is the temporal step size. CBC depletion was estimated using an exponential survival model based on experimentally determined lymphocyte radiosensitivity [23]. For $F_{n}\geq 2$, the accumulated dose $d_{i}^{\left(F_{j-1}\right)}$was reset to zero at the beginning of each fraction, assuming either complete repair of sublethal damage or cell death between fractions. A detailed description of the dynamic blood flow simulation is provided in [29]. For each fraction, b-DVHs, the irradiated blood-volume fraction $V_{D>0\;\mathrm{Gy}}$, and $D_{2\%}$were computed.

The total irradiated fraction of the blood pool was calculated after each treatment fraction. CBC depletion was estimated using the exponential survival model described above [23]. The fractions of the CBC receiving more than $V_{D>0.16\;\mathrm{Gy}}$ and $V_{D>0.3\;\mathrm{Gy}}$, corresponding to 5% and 10% CD4 depletion, respectively, were also quantified.

The relationship between CTV volume and technique-dependent CBC depletion was evaluated using a linear mixed-effects model. Treatment technique, CTV volume, and their interaction were included as fixed effects, while patient was included as a random intercept to account for the paired plans. The intersection between the fitted IMRT- and VMAT-associated curves was calculated as the CTV volume at which their predicted difference in CBC depletion was zero.

## 3 Results

**Fig. 3:**
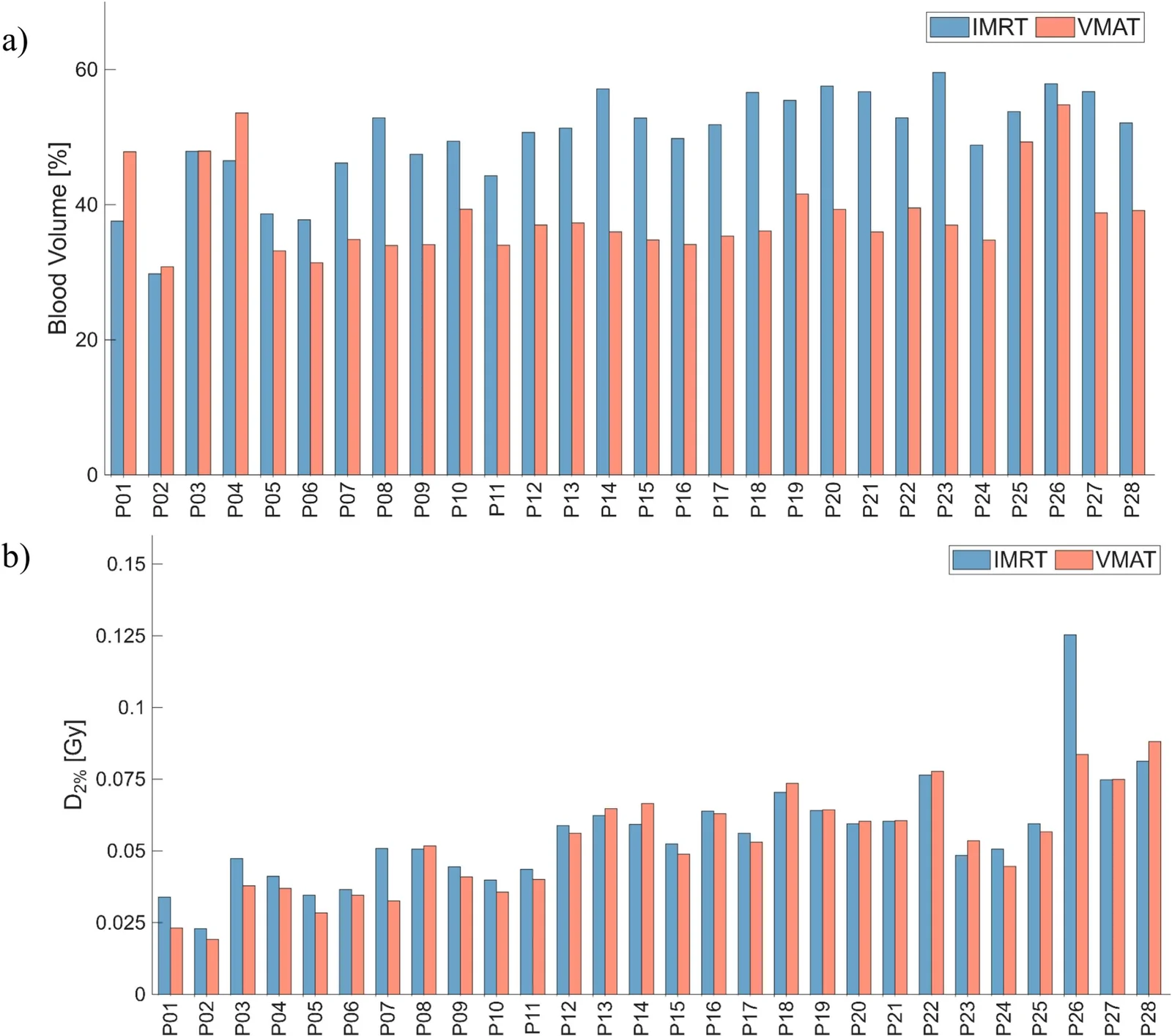
a) Side-by-side comparison of the fraction V_D>0mGy_ [%] and b) the D2% index [Gy] after a single treatment fraction of IMRT (gray) and VMAT (orange). The x-axis in all panels indicates patient numbers sorted as function of the CTV volume.

Across all 28 patients, the mean single fraction $V_{D>0\mathrm{Gy}}$ was 49.9% ± 7.2% with IMRT and 38.6% ± 6.3% with VMAT (μ±σ) (see Fig. 3a). The mean paired IMRT–VMAT difference was 11.3 percentage points ($p<10^{-5}$, paired *t*-test). The mean $D_{2\%}$ single fraction was 0.07 ± 0.02 Gy with IMRT and 0.06 ± 0.02 Gy with VMAT (Fig. 3b). The mean paired IMRT–VMAT difference was approximately 0.004 Gy (p=0.042, paired *t*-test).

**Fig. 4:**
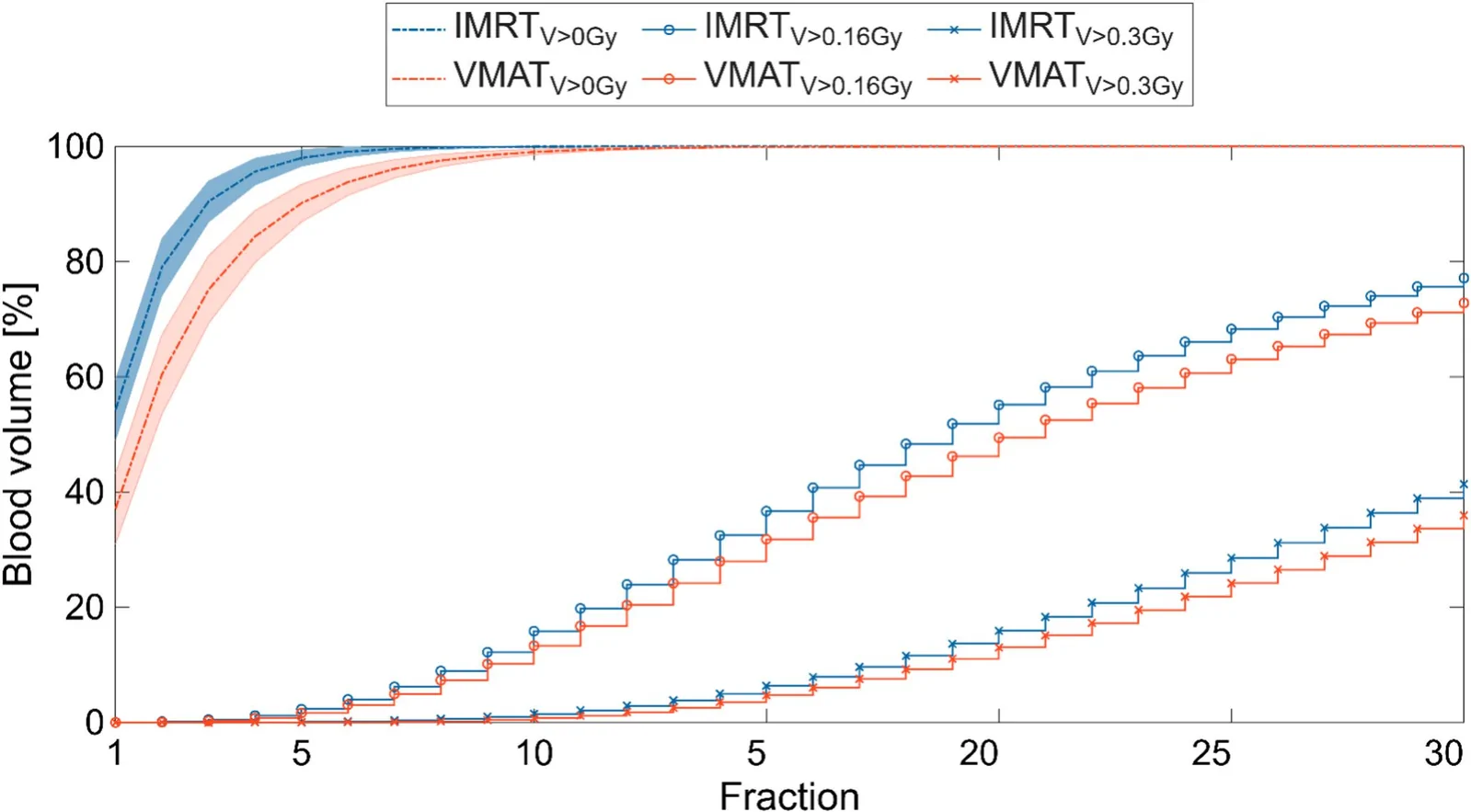
Mean fraction of CBCs receiving doses greater than 0 Gy, 0.16 Gy, and 0.3 Gy, accumulated over 30 fractions for IMRT and VMAT. Shaded areas represent ±1 standard deviation across the cohort.

After three fractions, the cumulative $V_{D>0\mathrm{Gy}}$ was 90% with IMRT and 77% with VMAT, corresponding to an absolute difference of 13 percentage points (Fig. 4). The cumulative $V_{D>0\mathrm{Gy}}$ reached approximately 95% after four fractions with IMRT and after seven fractions with VMAT. After 30 fractions, the accumulated $V_{D>0.16\mathrm{Gy}}$ was 76% with IMRT compared with 72% with VMAT, a difference of 4 percentage points. For accumulated $V_{D>0.3\mathrm{Gy}}$, the corresponding proportions were 40% and 35%, respectively.

**Fig. 5:**
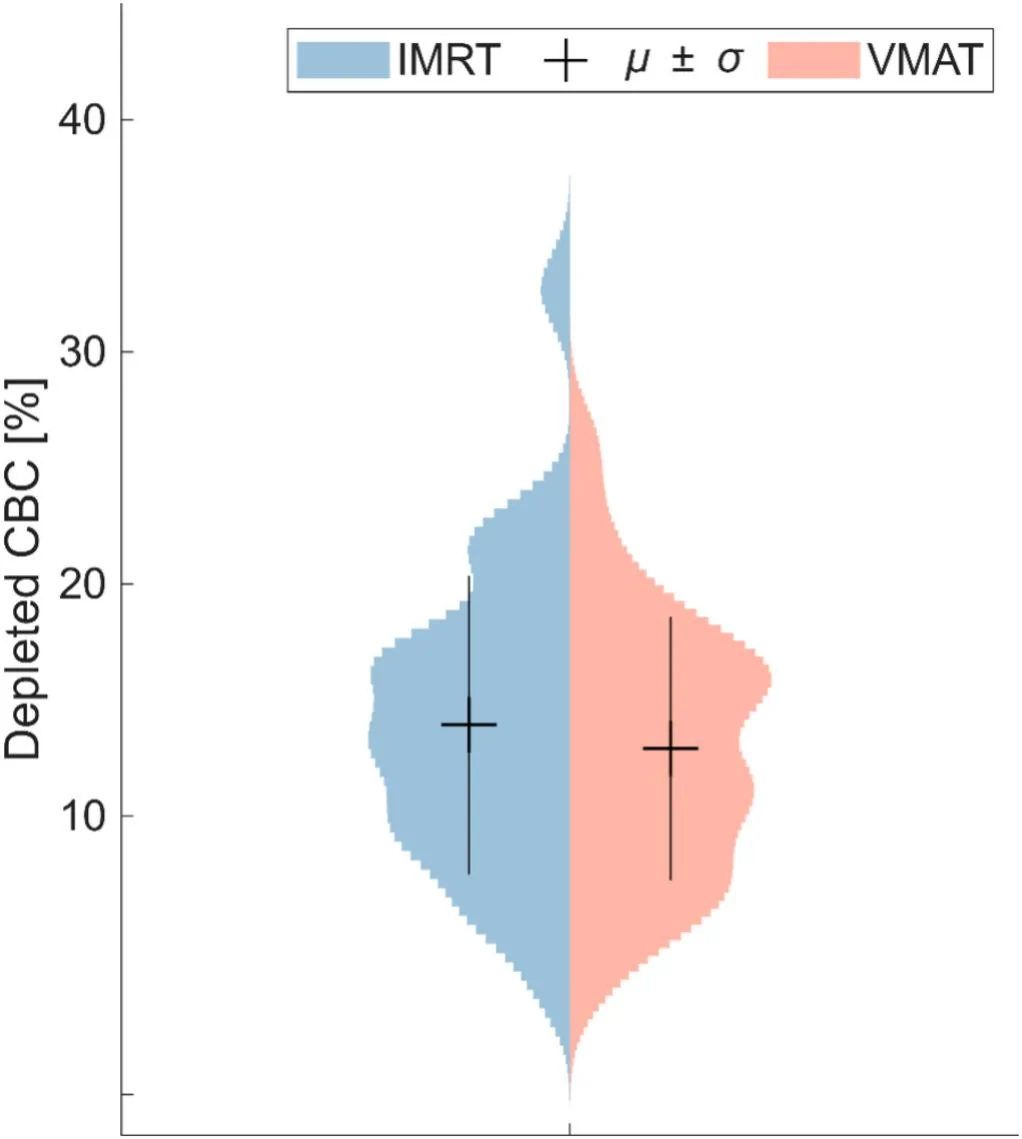
Split violin plot of estimated CBC depletion after 30 fractions across the cohort for IMRT (gray) and VMAT (orange). The y-axis shows the percentage of depleted CBC. Black crosses indicate the cohort mean ± standard deviation.

IMRT exhibited a longer depletion tail compared with VMAT (see Fig. 5). The mean CBC depletion was 13.9 ± 6.4% for IMRT and 12.8 ± 5.7% for VMAT. A paired comparison showed a mean difference of 1.0 ± 1.7% (IMRT – VMAT). The CI_95%_ [0.3%, 1.7%] did not include zero value, and a paired *t*-test showed a statistically significant difference (*p* = 0.003).

**Fig. 6:**
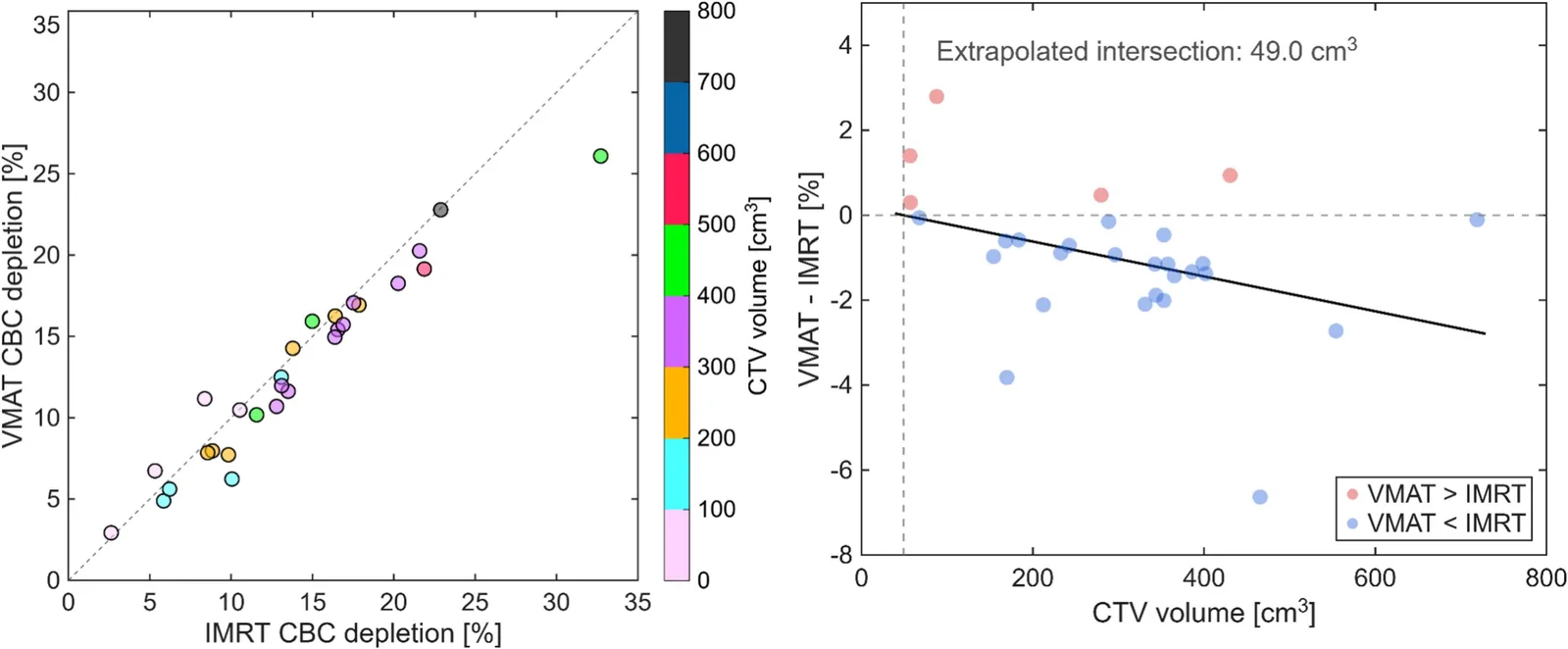
(left panel) Paired comparison of CBC depletion for IMRT and VMAT across all patients. The dashed line indicates equal depletion between techniques (y = x). CTV is color-coded in increments of 100 cm^3^. (right panel) Paired difference in simulated CBC depletion (VMAT - IMRT) as a function of CTV volume. Red points indicate higher depletion with VMAT, while blue points indicate lower depletion with VMAT. The solid black line represents the linear regression of the paired differences (p = 0.053), and the horizontal dashed line indicates equal depletion.

Of the 28 paired comparisons, 23 (82.1%) showed lower estimated depletion with VMAT, whereas five (17.9%) showed lower depletion with IMRT (Fig. 6). In an exploratory analysis, the CTV-by-technique interaction in the mixed-effects model indicated that the VMAT–IMRT difference decreased by 0.41 percentage points per 100 cm^3^ increase in CTV (interaction-term test, *p* = 0.043). A separate patient-level linear regression of the paired VMAT–IMRT differences against CTV produced a similar slope, although it did not reach statistical significance (regression-slope test, *p* = 0.053). The model-estimated zero-crossing was 49.0 cm^3^, below the smallest observed CTV of 57 cm^3^, and therefore represents an extrapolation beyond the observed range.

## 4 Discussion

This in silico study investigated how the spatial dose distributions and delivery dynamics of IMRT and VMAT affect dose accumulation in CBC. VMAT generally produces a broader low-dose bath but has a shorter and more continuous delivery. In contrast, IMRT uses more monitor units and has a longer segmented delivery, allowing more blood to recirculate through the treatment field [29]. These differences produced distinct b-DVHs. However, their competing effects resulted in only a small difference in overall simulated CBC depletion between the two techniques.

VMAT produced lower depletion than IMRT in 23 of 28 patients, although the magnitude and direction of the difference varied between patients. Estimated depletion increased with CTV volume for both techniques. The mixed-effects model also showed a technique-by-CTV interaction, suggesting that the relative sparing with VMAT may increase with CTV volume. However, the paired regression showed a similar trend, but it wasn't statistically significant (*p* = 0.053). Furthermore, the estimated zero-crossing of 49 cm^3^ was below the observed CTV range. The apparent CTV-dependent difference between the techniques should therefore be considered exploratory and requires further investigation in a larger cohort.

Although the mean paired difference was statistically significant, it was only approximately 1 percentage point, and its clinical relevance remains uncertain. A previous assessment found that intersubject variation in cerebral vasculature produced a standard deviation of approximately 11% of the mean predicted depletion [37], indicating that the observed technique-related difference was small relative to this variability. Using the same vascular model for both plans strengthened the paired comparison but did not reduce uncertainty in the absolute depletion estimates or in small differences for individual patients. Moreover, no validated minimum clinically important difference or blood-dose constraint currently links estimated CBC depletion to clinical lymphopenia. Nevertheless, minimizing unnecessary immune-cell exposure remains a reasonable objective, particularly in GBM, which is characterized by limited intratumoral T-cell infiltration and an immunosuppressive microenvironment [39], [40]. Several approaches, including sparing uninvolved brain tissue, hypofractionation, and proton therapy, have therefore been proposed to reduce radiation-induced lymphopenia [41], [42], [43]. However, the present results do not establish a clinically meaningful advantage for either IMRT or VMAT, as the model estimates only direct radiation-induced depletion and does not account for temozolomide, the disease itself, baseline CBC counts, or other clinical factors.

The systemic circulation component of the BFM was benchmarked against measured dilution curves, but this validates circulation kinetics rather than the predicted CBC depletion. Serial absolute lymphocyte count measurements were not available for the present cohort, preventing direct patient-specific validation against the simulations of this study. Published GBM studies reported reductions in absolute lymphocyte counts of approximately 35%–59% following radiotherapy with concurrent temozolomide [21], [44], [45], [46]. These clinical data provide context but cannot directly validate the present radiation-only estimates resulting from simulated results. The estimated depletion range of 2.6%–32.7% was lower than the clinically observed relative CBC reductions reported after chemoradiotherapy, as the model did not account for concurrent temozolomide, baseline CBCs counts, disease-related effects, or other clinical factors. Prospective studies incorporating serial absolute lymphocyte counts measurements are therefore required to assess the predictive accuracy of the proposed simulation model.

All plans met the prespecified target criteria and were considered clinically acceptable by two radiation oncologists. Thus, differences in CBC exposure were unlikely to result from systematic differences in plan quality. Because each patient contributed both an IMRT and a VMAT plan, the imbalance in the clinically delivered techniques did not create unequal comparison groups; it only determined which plan was delivered clinically and which was generated retrospectively.

The cohort was limited to 28 patients, which restricts the precision and generalizability of the findings despite the paired design. The glioma cohort was predominantly composed of patients with GBM but also included four patients with astrocytoma and one with oligodendroglioma. Histology was not an input to the BFM and is therefore not expected to directly affect the paired dosimetric comparison. However, differences in biological and immune response between glioma subtypes may affect clinical CBC depletion. The small number of non-GBM cases did not allow a meaningful subgroup analysis.

## CRediT authorship contribution statement

**Abdelkhalek Hammi:** Writing – original draft, Visualization, Validation, Software, Resources, Methodology, Investigation, Funding acquisition, Formal analysis, Data curation, Conceptualization. **Nadya Shusharina:** Writing – review & editing, Validation, Supervision, Methodology, Formal analysis, Data curation, Conceptualization. **Eleni Gkika:** Writing – review & editing, Validation, Supervision, Resources, Investigation, Formal analysis. **Youness Nour:** Writing – review & editing, Validation, Software, Methodology, Investigation, Formal analysis, Data curation, Conceptualization.

## Declaration of competing interest

The authors declare that they have no known competing financial interests or personal relationships that could have appeared to influence the work reported in this paper.
