# Supplementary material for: Impact of dynamic beam delivery of intensity-modulated radiation therapy and volumetric modulated arc therapy on circulating immune cell exposure in glioma
Source: Phys Imaging Radiat Oncol. 2026 Aug 25;41:101070. doi: 10.1016/j.phro.2026.101070 (PMC13580052; doi:10.1016/j.phro.2026.101070)
Supplement: Supplementary file 1 [file mmc1.pdf]

Supplementary material

Table S1: Overview over the different treatment planning scenarios

Case #	CTV size [cm^3]	Initial Clinical Modality
1	56.6	VMAT
2	56.9	IMRT
3	67.4	VMAT
4	87.8	IMRT
5	154.3	IMRT
6	168.3	VMAT
7	169.7	VMAT
8	183.5	VMAT
9	212.4	VMAT
10	232.6	IMRT
11	242.6	VMAT
12	279.7	VMAT
13	288.6	IMRT
14	296.2	IMRT
15	331.2	VMAT
16	342.5	VMAT
17	343.9	VMAT
18	353	VMAT
19	353	IMRT
20	357.6	IMRT
21	365.2	VMAT
22	386.1	IMRT
23	398.8	VMAT
24	401.8	VMAT
25	430.5	IMRT
26	465.4	VMAT
27	554.2	IMRT
28	719	IMRT
